# Microsurgical Strategies after Free Flap Failure in Soft Tissue Reconstruction of the Lower Extremity: A 17-Year Single-Center Experience

**DOI:** 10.3390/jpm12101563

**Published:** 2022-09-22

**Authors:** Felix Struebing, Lingyun Xiong, Amir K. Bigdeli, Yannick Diehm, Ulrich Kneser, Christoph Hirche, Emre Gazyakan

**Affiliations:** 1 Department of Hand-, Plastic and Reconstructive Surgery, BG Trauma Center Ludwigshafen, Heidelberg University, 67071 Ludwigshafen, Germany; 2 Department of Plastic Surgery, Union Hospital, Tongji Medical College, Huazhong University of Science and Technology, Wuhan 430022, China; 3 Department of Plastic, Hand- and Reconstructive Microsurgery, Hand Trauma- and Replantation Center, BG Trauma Center Frankfurt am Main, Affiliated Hospital of Goethe University Frankfurt, 60389 Frankfurt, Germany

**Keywords:** lower extremity, free flap, flap failure, secondary free flap, tertiary free flap, orthoplastic, limb salvage

## Abstract

**Background:** There is no clear consensus on the optimal surgical strategy for providing safe coverage in salvage free flap surgery after total free flap failure. **Methods:** A retrospective study was conducted to evaluate patients with total failure of the primary free flap in lower extremity reconstruction between 2000 and 2017. **Results:** In a cohort of 1.016 patients, we identified 43 cases of total flap failure (4.2%). A total of 30 patients received a salvage free flap with a success rate of 83.3% (25/30). One patient received a secondary salvage free flap. Overall limb salvage after primary free flap loss was 83.7% (36/43). **Conclusions:** Microsurgical management of free flap loss in the lower extremity is challenging and requires a decisive re-evaluation of risk factors and alternative strategies. This should include reconsidering the flap choice with a tendency towards traditional and safe workhorse flaps, a low-threshold switch to different recipient vessels, including arteriovenous (AV) loops, bypasses (especially in case of venous insufficiency) and back-up procedures, such as negative pressure wound therapy or dermal regeneration templates with skin grafting in cases of lower demand and critically ill patients. We derived one suggestion from our previous practice: replacing perforator flaps with axial pattern flaps (“safe workhorses”).

## 1. Introduction

Despite significant advances in microsurgical techniques and instruments, there are still patients suffering from inevitable flap failure. This represents a major complication in microsurgical lower extremity reconstruction, increasing the risk for amputation and thereby, resulting in decreased functional capacity, diminished quality of life, and likely a higher mortality rate [1]. Although the existing literature provides an appropriate level of evidence on flap type, donor site, and recipient vessel choice, the surgical strategy for microsurgical salvage procedures after free flap failure in lower extremity reconstruction remains controversial and limited [2,3].

Many factors must be considered in the planning of reconstructive salvage procedures, including the reevaluation of the defect and the assessment of available recipient vessels, donor-site morbidity, and additional procedures, such as vein grafting or the placement of arteriovenous (AV) loops. In addition, the reconstructive surgeon should consider the whole spectrum of reconstructive options [4,5,6,7]. Secondary amputations must also be kept in mind and weighed against limb salvage, especially when an insensate and nonfunctional limb might be the ultimate reconstructive result.

Evidence-based safety precautions and secondary free flap strategies in lower extremity reconstruction after flap failure remain limited and inconsistent. Therefore, microsurgical salvage procedures are still considered controversial among some reconstructive surgeons [2,8,9].

The aim of the present study is to provide more evidence on the management and strategies of flap failure in lower extremity reconstruction using secondary free flaps. In this context, we analyzed a cohort of 1.016 consecutive lower extremity free flaps to provide recommendations for salvage reconstruction using free tissue transfer.

## 2. Patients and Methods

A retrospective review of a prospectively maintained lower extremity reconstruction database at the BG Trauma Center Ludwigshafen, Heidelberg University, was undertaken. The local ethics committee (Mainz, Germany) approved the study. It was carried out in accordance with the Declaration of Helsinki. Patients who met the inclusion criteria were selected for further analysis. The inclusion criteria were: (a) soft tissue defect in the lower extremity with intact plantar sensitivity, (b) age ≥ 18 years, (c) autologous free tissue transfer, (d) total failure of the primary free tissue transfer, (e) complete medical records. The database contained cases from April 2000 to December 2017. Medical records of each patient were reviewed and data on the following parameters were collected: age, sex, etiology of the defect, location of the defect, result of preoperative angiography, recipient vessels, microsurgical anastomosis technique, type of free flaps used, postoperative complications and outcomes, and amputation. Postoperative complications included arterial and venous thromboses, venous insufficiencies, wound complications, hematomas, and flap loss.

### 2.1. Microsurgical Revision Strategy

In the present study, all patients were treated with the same perioperative and surgical management protocols. All microsurgical procedures were performed as previously described [10,11]. Usually, the same surgeon performs the secondary or tertiary reconstructions. To ensure the maximum level of safety, an experienced senior reconstructive surgeon participated in all revision cases. In case of vascular compromise following free tissue transfer, emergent takeback was performed. This always included exploration of recipient and donor vessels and anastomotic revision in warranted cases. In case of extensive thrombosis or suspicion of in-flap thrombosis, a thrombolytic therapy was performed. Postoperatively, patients with increased procedure related comorbidities or prolonged operative time due to technical complexity were admitted for intensive care unit (ICU) monitoring [12].

### 2.2. Postoperative Monitoring

All free tissue transfers were monitored hourly for the first 48 h after surgery, followed by an evaluation every two to four hours for five days by clinical assessment of capillary refill, turgor, and color. In addition, handheld Doppler was used when required.

All patients received postoperative anticoagulation. Low-molecular-weight heparin was administered twice daily in a dosage of 0.4–0.6 mL for five days. In cases of hypercoagulable disorders, patients received a continuous infusion of high-molecular-weight heparin under repeated activated partial thromboplastin time (aPTT) controls.

### 2.3. Statistical Analysis

Normally distributed continuous variables were presented with mean ± SD or N in % and analyzed using Student’s t test or Wilcoxon rank sum test. Categorical variables were compared using Chi2, Fisher’s exact test or Wilcoxon rank sum test. All statistical analyses were two-tailed and values of *p* < 0.05 were considered significant. Statistical analyses were performed using SPSS 19.0 (SPSS Inc., Chicago, IL, USA).

## 3. Results

### 3.1. Patient Characteristics

A total of 1.016 free tissue transfers for primary lower extremity reconstruction were performed in our department during the study period. In this cohort, 588 fasciocutaneous flaps (57.8%) and 428 muscle flaps (42.2%) were utilized. Forty-three (4.2%) of the 1.016 free tissue transfers failed completely. The study cohort included eleven women (25.6%) and 32 men (74.6%). The mean age of the patients was 48.6 ± 17.1 years. There were 14 patients with adiposity (BMI > 30 kg/m^2^), eleven active smokers (25.6%), ten cases of diabetes (23.2%), eight patients suffering from arterial hypertension (18.6%) and four cases of coagulopathies (9.3%).

The most common causes of soft tissue defect in our study cohort were trauma in 18 cases (41.9%), chronic posttraumatic osteomyelitis (CPTO) with infection in 17 cases (39.5%), and chronic/non-healing wounds in four cases (9.4%). The mean time between trauma and free tissue transfer was 26.2 ± 19.8 days. The remaining causes were burn injuries (*n* = 2; 4.6%), peripheral arterial occlusive disease (*n* = 1; 2.3%) and ischemic necrosis of the lower extremity after arterial thrombosis (*n* = 1; 2.3%). The mean interval from trauma to reconstruction was 23.3 days ± 20.3 days.

Regarding defect localization, twenty-eight defects (65.1%) were in the lower leg, nine defects (20.9%) in the foot, three defects (7.0%) around the ankle, and three defects (7.0%) around the knee. The mean wound size was 224.7 cm^2^. Preoperative computed tomographic angiography (CTA) of the lower extremity was performed in all patients before primary free flap reconstruction. In 17 patients (39.5%), vascular abnormalities were noted on CTA. CTA revealed in seven patients (16.3%) merely one run-off artery in the lower leg and in another ten patients (23.2%) two run-off arteries. The remaining patients (*n* = 26; 60.5%) demonstrated normal vascular anatomy of the lower leg (Table 1). The average follow-up duration was 25.7 months (±26.8 months).

### 3.2. Failed Primary Reconstructions

Our study cohort of forty-three failed primary reconstructions of the lower extremity included nineteen anterolateral thigh (ALT) flaps (44.2%), nine myocutaneous latissimus dorsi (LD) flaps (20.9%), five gracilis muscle flaps (14.7%), two combined LD–parascapular flaps (5.9%), and another two lateral arm free flaps (5.9%). The remaining six free tissue transfers consisted of each one (2.3%) rectus abdominis muscle free flap, free fibula osteoseptocutaneous flap, superficial circumflex iliac artery flap, serratus muscle flap, parascapular (PSC) flap, and radial forearm flap. The posterior tibial artery (PTA) was used most frequently as the recipient artery (18 cases, 41.9%), followed by the anterior tibial artery (ATA) in 15 cases (34.9%), and the superficial femoral artery (SFA) in 2 cases (4.6%), respectively. In case of unsuitable recipient arteries, eight AV loops (18.6%) and four interpositional vein grafts (9.3%) were performed. The type of anastomosis was end-to-side in 26 cases (60.5%) and end-to-end in 17 cases (39.5%), respectively. In most cases, a single venous anastomosis was performed in end-to-end fashion (*n* = 41; 94.4%); multiple venous anastomoses were performed in two cases (4.6%).

The most flaps were lost in the first 24 h after surgery (*n* = 20, 46.5%), ten flaps failed 24–72 h after the free flap transfer (23.3%) and thirteen flaps were lost more than 72 h after surgery (30.2%). Fourteen out of forty-three free tissue transfers (32.6%) were lost due to arterial thrombosis. Further flap failures occurred because of venous thrombosis in five cases (11.6%) and combined arterial and venous thrombosis in six cases (13.9%), respectively. Two occlusions of AV loops (4.6%) resulted in flap failure. In three cases (6.9%), microvascular complications occurred perioperatively. In these cases, flap failure was due to arterial thrombosis and disseminated in-flap thrombosis (no-reflow phenomenon). The development of severe infection led to flap failure in five cases (16.3%). The postoperative no-reflow phenomenon was observed in three cases (7.0%), probably because of in-flap thrombosis. There were more failed cases of muscle flaps (*n* = 24; 55.8%) than failed fasciocutaneous flaps (*n* = 19; 44.2%). Table 1 shows the data for the primary reconstructions.

### 3.3. Salvage Reconstruction and Postoperative Surgical Outcome

Most free flap failures were salvaged with a second free tissue transfer (*n* = 30; 69.8%). The distribution between muscle flaps (*n* = 16; 53.3%) and fasciocutaneous flaps (*n* = 14; 46.7%) was similar. Salvage reconstructions were performed with LD flaps (*n* = 15; 50.0%), ALT flaps (*n* = 6; 20.0%), PSC flaps (*n* = 6; 20.0%), radial forearm flaps (*n* = 2; 56.7%) and one combined LD-PSC flap (3.3%). Interestingly, thirteen of eighteen (72.2%) failed fasciocutaneous flaps were salvaged with muscle flaps and five of nine (55.5%) failed muscle flaps were salvaged with fasciocutaneous flaps, respectively. Secondary defect coverage was performed with dermal regeneration templates and split-thickness skin grafting in four cases (9.3%). In addition, one patient (2.6%) was transferred to a hospital near his place of residence on personal request with NPWT for wound healing by secondary intention, and one patient (2.6%) received a perforator propeller flap. In this cohort, limb salvage could not be achieved in seven patients (16.3%) who subsequently received an amputation. Five patients (71.4%) received knee disarticulations and two transtibial amputations (28.6%). In all cases, the reason for amputation was an extended bone exposure. This was made more difficult by persistent bone or soft tissue infections in three cases and poor one vessel run-off in the remaining four patients.

The recipient artery was changed in ten of thirty cases (33.3%). Among these ten cases, six cases (60%) received AV loops, and in four cases (40%), another distal lower leg artery was chosen, respectively. The type of arterial anastomosis changed in 14 cases (46.7%). In eleven out of fourteen cases (78.4%), it was changed to end-to-end anastomoses. Among these cases, four AV loops (28.6%) were established to move even further out of the zone of injury. Arterial anastomosis was changed to end-to-side in three cases (21.4%). The mean time between primary and secondary microsurgical reconstruction was 16.3 ± 11.6 days (range: 3–55 days). More detailed information is presented in Table 2.

Secondary limb salvage was successful in 31 cases (72.1%). Flap failure rate after salvage free flap was 16.7%. Flap failure occurred in four out of thirty secondary free flaps (13.3%) postoperatively, whereas one secondary LD flap (3.3%) suffered from severely compromised perfusion during intraoperative dissection. Therefore, the free flap was not transferred, and the defect was covered with NPWT. Three flap failures (10.0%) were caused by microvascular thrombosis, whereas the fourth free flap loss remained unclear. In these five secondary failed free flaps, thrombophilia screening resulted in negative results. Table 2 shows the sequence of reconstructive procedures performed in each patient. Table 3 depicts the results of the salvage reconstructions.

### 3.4. Secondary Salvage Reconstruction and Postoperative Surgical Outcome

Secondary salvage procedures were performed in 5 out of 30 cases (16.6%). One patient was transferred to a home hospital on his personal demand with NPWT before further salvage procedures were initiated. Three out of the four patients (75%) were not suitable for a tertiary free flap transfer anymore because of single vessel run-off or concurrent infection. However, no thrombophilia was found in these cases. After debridement of the flap and interim NPWT, a delayed local random pattern flap and a delayed reversed sural flap were performed in two cases. One case received dermal regeneration templates and split-thickness skin grafting. Finally, one patient (20%) received a tertiary free LD flap transfer (end-to-side anastomosis to the posterior tibial artery) since both ALT flaps were used in the primary and salvage reconstruction. All four patients were successfully treated. The overall limb salvage rate was 83.7% (36/43 cases). Table 4 contains information on the secondary salvage procedures.

## 4. Discussion

Microsurgical free tissue transfers in lower extremity reconstruction proved to be reliable and successful procedures over the past decades. A recent meta-analysis of 1397 free tissue transfers for lower extremity reconstruction calculated a flap failure rate of 6% [13]. However, the risk of free flap failure is omnipresent and, with it, the risk of secondary amputation. Previous reports demonstrated significant long-term mortality rates between 61 and 71% after lower limb amputation [14,15]. However, within the scope of the lower extremity assessment project (LEAP) multicenter study, various studies could demonstrate identical functional outcomes, return to work rates, and scores for the sickness impact profile after limb salvage or amputation [14,15,16]. In addition, Harris and colleagues reported a higher rate of complication and rehospitalization in the limb salvage group than in the amputation group at 2 years of follow-up [17]. Previous studies on the hand reported that patients would almost always favor limb salvage over amputation [18,19].

At our center, we trust in a multidisciplinary approach for tailoring an individualized reconstructive approach for each patient. The formalized collaboration between orthopaedic, plastic, and vascular surgeons aims at improving surgical outcome and patient care. We utilize weekly meetings of trauma/orthopedic, vascular, and plastic surgeons together with physical therapists to evaluate cases that require multidisciplinary treatment. Boriani and colleagues could demonstrate that an orthoplastic approach improved outcome measures in patients with severe open tibial fractures as opposed to conventional orthopedic care [20]. In a recent retrospective review of patients undergoing flap-based limb salvage for combat-related extremity trauma, Hoyt and colleagues report a decrease in flap failures when an orthoplastic approach is implemented [21]. In our center, the multidisciplinary approach is well accepted and implemented, leading to a compliance rate of 92% [22]. However, as proposed by Azoury et al., treatment protocols must be questioned on a constant basis and new guidelines must be implemented to ensure successful limb salvage [23]. Nevertheless, flap failure is an evident threat and the consequential salvage procedures should be subject to a similar approach. The procedures can range from simple re-anastomoses over vein grafts to secondary flaps utilizing the entire microsurgical armamentarium for improved flap-based limb salvage outcomes.

In our previous study on 581 lower extremity free flap reconstructions, we reported a failure rate of 5.9% [24]. Prior studies displayed total flap failure rates in lower extremity reconstructions between 6.9% and 8.5% [25,26]. A recent meta-analysis overlooking 862 flaps reported an overall free flap failure rate of around 9.6% [27]. Notably, total flap failures were similar between muscle and fasciocutaneous flaps. Cho and colleagues described their experience among muscle and fasciocutaneous free flaps in acute trauma and chronic traumatic sequelae [28]. Their two subgroups did not differ in total flap failure rates. Stranix and colleagues demonstrated similar results over a forty-year period [29]. Interestingly, they reported an earlier and more frequent take-back of fasciocutaneous free flaps, however, with 9.4 times higher salvage rates than muscle flaps. A more recent meta-analysis on lower extremity salvage in the setting of osteomyelitis also revealed that either muscle or fasciocutaneous flaps can be utilized safely with comparable results [30].

In our series, we could demonstrate a similar free flap failure rate of 4.2% (*n* = 43) in 1.016 lower extremity free flap reconstructions. Compared to the data from a review of the literature by Lineaweaver and colleagues, we decided to opt for a second free flap as a limb salvage procedure twice as often [31]. In our opinion, the decision on whether to attempt another reconstruction or amputate should be decided by exclusion. All patients should be scheduled for a secondary microsurgical reconstruction, except those who have comorbidities so severe that they prohibit a secondary major surgery. This is consistent with the recommendations of many other high-volume microsurgical institutions [2,32,33]. The reconstructive possibilities are always discussed openly with the patient, and our recommendations, as well as all other options, are presented and evaluated, including reconstruction, amputation, or conservative management.

We performed 30 secondary free flaps (69.8%) with a success rate of 83.3%. Culliford and colleagues reported a secondary free flap success rate of 63% [25]. However, it must be noted that they attempted a secondary free flap as a salvage procedure in 16% of cases. Previous studies substantiated the rightness of secondary free flaps with successful results [8,34,35,36]. Hallock demonstrated 16 total flap failures in 298 free perforator flaps over a 10-year period (5%) [37]. Of these, 11 patients received secondary free flaps (68.8%) with a success rate of 72.7%. Patients received four muscle flaps and seven perforator free flaps.

We concluded one general recommendation from our previous practice: replacing perforator flaps with axial-pattern flaps (“safe workhorses”). In our data, secondary free LD flaps were used in most cases (57.9%) instead of an ALT free flap. Axial-pattern flaps provide a consistent anatomy, easier dissection, and mostly large diameter pedicles and are therefore our first choice for salvage free flap reconstructions. Nevertheless, it must be noted that other authors reported successful usage of secondary perforator flaps after flap failure of a primary perforator flap with success rates of up to 100% [8].

In our cohort, one patient in whom the secondary free flap failed, was successfully treated with a tertiary free flap (1 out of 5, 20%). A more recent study by Moratin and colleagues showed an overall flap success rate of approximately 89% in patients receiving head and neck reconstructions with consecutive free flaps [38]. They retrospectively analyzed 996 free flaps with 220 reconstructions using 2 to 6 flaps in 189 patients, stating that prior flap loss is not prognostic of the success of back-to-back reconstructions. They only identified diabetes mellitus as a predictor of free flap failure.

Since Godinas’ seminal work in 1986 presenting his experiences with early lower extremity reconstruction, which lay ground the “Godina principles” [39], the timing of lower extremity reconstruction was a topic of debate. The advent of negative pressure wound therapy allowed an extension of the previously requested time frame of three days to soft tissue reconstruction [40]. Nonetheless, a timely soft tissue coverage should be the goal. In our experience, this can be difficult in the setting of secondary referrals that in many cases come with a long delay. In this context, the findings of our study add evidence to the requirement of a timely soft tissue reconstruction. Only two out of eighteen acute trauma cases were reconstructed in the first seven days following the trauma. This could be seen as further evidence that suggests the need for a timely soft tissue reconstruction.

In our previous study, we showed that perforator flaps have an increased risk of microvascular complications when anastomosed to an AV loop. This is most likely based on the assumption of an increased flow resistance of the small-caliber perforators [41]. Therefore, we recommend the use of fasciocutaneous axial pattern or muscle flaps if an AV loop is needed. However, Momeni and colleagues reported high success rates of perforator flaps anastomosed to AV loops in a matched-pair analysis of lower extremity reconstruction [42].

In case of microvascular thrombosis, choosing a new recipient vessel (e.g., deep to superficial venous system) and alternating the anastomotic technique (e.g., end-to-end anastomosis instead of end-to-side) needs to be considered. In vessel depleted extremities, AV loops and vein grafts should always be considered [43]. Additionally, using a flap with a long pedicle, such as the LD flap, may be considered to facilitate an anastomosis more proximal to the zone of injury in trauma patients. A trend or recommendation to change the recipient vessel or anastomosis type could not be deducted from the study data.

A total of four patients (9.3%) in our cohort were successfully treated with de-escalation of the reconstructive ladder, including NPWT or NPWTi and skin grafting with or without dermal substitutes. In complex wound situations NPWT may allow for wound granulation, either for skin grafting or in preparation for a secondary free flap. In cases where an additional free flap might not be feasible, skin grafting with or without a dermal regeneration template and with or without preceding NPWT or NPWT with wound irrigation (NPWTi) could be an appropriate alternative [44].

A thorough work-up of patient-specific risk factors for flap loss should be undertaken. We recommend considering peripheral bypasses and single- or two-step AV loops in the strategic reevaluation. In complex cases or cases of suspected peripheral arterial occlusive disease, a vascular surgeon should be contacted in the planning of the primary case—not just in the case of primary failure. A secondary angiography is helpful to detect unforeseeable changes to the vasculature after free flap loss, which can be completed by phlebography or Duplex ultrasound of the venous system in selected cases [7,45,46]. In addition, an extended thrombophilia screening, including standard coagulation parameters and rotational thromboelastometry (ROTEM) and genetic analysis, is recommended to detect changes in posttraumatic cases. If these analyses reveal a pro- or anti-coagulatory disorder, a specialized hematologist should be involved. Orthoplastic principles should be applied and the critical debate about whether amputation or limb salvage is preferable deserves special attention.

The presented study comes with inherent limitations. While we were able to present the experiences of more than 1.000 free flaps in the lower extremity reconstructions, the study cohort is still relatively small, with only 43 patients. Its retrospective nature and small sample size expose the study to observer and selection bias. Furthermore, final ambulatory status was not assessed, which may also confound final clinical decision-making. The fact that multiple surgeons were involved in the microsurgical procedures of this cohort potentially led to a performance bias. Despite these limitations, the results presented in this study include a comparably high number of salvage free flaps in lower extremity reconstruction and might help in the clinical decision-making process. Our findings warrant the use of consecutive salvage procedures when flap loss and thereby associated limb salvage failure is at risk.

## 5. Conclusions

The microsurgical management of the loss of a primary free flap in the lower extremity is challenging and demands a concise re-evaluation of risk factors and alternative strategies. An additional free flap reconstruction should be attempted if the patient does not have significant comorbidities that prohibit another major surgery. Concluding from our previous practice in a series of 1.016 lower extremity free flap reconstructions, we recommend switching to an axial pattern free flap in cases of perforator flap failure. After free primary flap failure, we obtained a limb salvage rate of 83.7%.

## Figures and Tables

**Table 1:** Patient characteristics and data on the failed primary microsurgical reconstruction.

Patient No.	Age/Sex	Indication	Location of Defect	No. of Patent Arteries in Lower Leg	First Free Flap	Recipient Artery	Recipient Vein	Anastomosis A/V	VG	Cause of Failure
**1**	10/M	Trauma	Lower leg	2	LD-PARA	ATP	VC	EE/EE	--	A. Thrombosis
**2**	39/M	Infection	Foot	2	Lateral uparm	ATA	VS	EE to loop/EE	AV Loop	A. Thrombosis
**3**	59/M	Burn	Malleolus	3	ALT	ATA	VC	ES/EE	--	Infection
**4**	27/M	Trauma	Knee	1	LD	AFs	VS	ES/EE	AI	Intraop failure, A. Thrombosis
**5**	76/F	Infection	Dis. lower leg	3	LD	ATP	VS	ES/EE	--	MRSA infection
**6**	85/F	PAOD	Dis. lower leg	1	Radial forearm	ATP	VC	EE to loop/EE	AV Loop	A. Thrombosis
**7**	48/M	Infection	Foot	3	ALT	ATA	VC	EE/EE	--	V. Thrombosis
**8**	52/M	Infection	Lower leg	3	ALT	ATP	VS	ES/EE	--	A. Thrombosis
**9**	43/M	Trauma	Dis. lower leg	3	LD	ATA	VC	ES/EE	--	A. Thrombosis
**10**	33/F	Infection	Dis. lower leg	2	Gracilis	ATP	VC + VS	ES/EE	AI	A. et V. Thrombosis
**11**	44/M	Trauma	Mid and dis. lower leg	2	LD	ATP	VC	EE/EE	--	A. insufficient, infection
**12**	48/M	Infection	Lower leg	3	ALT	ATP	VS	ES/EE	--	V. Thrombosis
**13**	70/F	Infection	Lower leg	2	ALT	ATP	VC	ES/EE	--	Ischaemia because of intraop injury of perforator
**14**	59/M	Trauma	Mid lower leg	1	ALT	ATP	VC	ES/EE	--	Ischaemic flap, no thrombosis in pedicle
**15**	69/M	Infection	Dis. lower leg	3	ALT	ATA	VS	ES/EE	--	A. et V. Thrombosis
**16**	56/M	Infection	Dis. lower leg	2	Gracilis	ATP	VC	EE/EE	--	In-flap thrombosis
**17**	54/F	Trauma	Malleolus	2	Gracilis	ATP	VS	ES/EE	--	V. Thrombosis
**18**	17/M	Burn	Lower leg	2	ALT	ATA	VC	ES/EE	--	A. Thrombosis
**19**	24/M	Trauma	Lower leg	2	LD-PARA	ATP	VS	ES/EE	--	A. et V. Thrombosis
**20**	43/M	Infection	Lower leg	2	LD	Apop	VS	ES/EE	--	Infection
**21**	59/M	Trauma	Malleolus	2	LD	ATA	VC	ES/EE	--	A. et V. Thrombosis
**22**	57/M	Trauma	Foot and malleolus	2	ALT	ATA	VC	ES/EE	--	Infection
**23**	37/M	Infection	Lower leg	1	Lateral uparm	Apop	VS	EE to loop/EE	AV Loop	A. Thrombosis
**24**	52/F	Trauma	Foot	2	Gracilis	ATP	VC	ES/EE	--	Infection
**25**	55/M	Trauma	Lower leg	2	ALT	Afs	VC	EE to loop/EE	AV Loop	Iscaemic damage after Thrombosis of AV Loop
**26**	37/M	Infection	Lower leg	1	ALT	Afs	VS	EE to loop/EE	AV Loop	A. et V. Thrombosis of AV Loop
**27**	48/M	Trauma	Malleolus	3	ALT	A. genicularis descendens	VS	EE to loop/EE	AV Loop	V. Thrombosis
**28**	75/F	Wound healing disorder	Dis. lower leg	1	ALT	ATA	VS	ES/EE	--	Intraop failure, no reflow
**29**	49/M	Infection	Dis. lower leg	2	FIBU	ATP	VC	EE to loop/EE	AV Loop	A. Thrombosis
**30**	47/M	Wound healing disorder	Dis. lower leg	2	ALT	ATA	VC	EE/EE	--	A. Thrombosis
**31**	65/F	Trauma	Dis. lower leg	1	RA	ATP	VS	ES/EE	--	A. et V. Thrombosis
**32**	47/M	Wound healing disorder	Mid and dis. lower leg	3	LD	ATP	2VC	ES/EE	--	In-flap thrombosis, APC-resistance
**33**	49/M	Infection	Foot	2	Gracilis	ATA	VC	ES/EE	--	A. Thrombosis
**34**	40/M	Infection	Dis. lower leg	1	ALT	Afs	VS	EE to loop/EE	AV Loop	In-flap thrombosis
**35**	17/M	Trauma	Foot	3	Serratus	Apl	VC	EE/EE	--	Hematoma
**36**	36/W	Trauma	Foot	2	LD	ATP	VC	ES/EE	--	Insufficient venous drainage
**37**	57/M	Trauma	Foot	3	ALT	ATA	VC	EE/EE	--	A. Thrombosis
**38**	55/M	Trauma	Knee	2	Parascapular	Afs	VC	ES/EE	--	Hematoma
**39**	48/W	Wound Healing Disorder	Dis. lower leg	3	SCIA	ATA	VC	EE/EE	--	A. Thrombosis
**40**	16/M	Infection	Knee	3	ALT	ATA	VC	EE/EE	AI	A. Thrombosis
**41**	80/W	PAOD	Dis. lower leg	3	LD	ATP	VC	EE/EE	--	Hypoperfusion during systemic hypotension
**42**	38/M	Trauma	Malleolus	3	ALT	ATA	VC	EE/EE	--	A. Thrombosis
**43**	55/M	Infection	Dis. lower leg	2	ALT	Afs	VC	ES/EE	AI	A. et V. Thrombosis

PAOD: peripheral arterial occlusive disease; VG: venous grafting; LD: latissimus dorsi; PARA: parascapular; ALT: anterolateral thigh; RA: rectus abdominis; SCIA: superficial circumflex iliax artery flap; ATA: arteria tibialis anterior; ATP: arteria tibialis posterior; Afs: arteria femoralis superficialis; Apop: arteria poplitea; Apl: A. plantaris medialis; VS: vena superficialis; VC: venae comitantes; ES: end to side; and EE: end to end.

**Table 2:** Consecutive reconstructions.

Patient No.	Primary Free Flap	Salvage Free Flap/Reconstruction	Secondary Salvage Free Flap/Reconstruction
**1**	LD-PARA	Amputation	--
**2**	Lateral uparm	Split thickness skin graft	--
**3**	ALT	ALT	--
**4**	LD	Amputation	--
**5**	LD	Parascapular	--
**6**	Radial forearm	Amputation	--
**7**	ALT	Parascapular	--
**8**	ALT	LD	--
**9**	LD	Matriderm + skin graft	--
**10**	Gracilis	LD	--
**11**	LD	LD	--
**12**	ALT	Parascapular	--
**13**	ALT	LD	--
**14**	ALT	ALT	--
**15**	ALT	LD	--
**16**	Gracilis	LD	--
**17**	Gracilis	RA	--
**18**	ALT	LD	--
**19**	LD-PARA	Amputation	--
**20**	LD	Amputation	--
**21**	LD	Amputation	--
**22**	ALT	LD	--
**23**	Lateral uparm	Transfer to hospital closer to the patients place of residence	--
**24**	Gracilis	Radial forearm	Delayed reversed sural flap
**25**	ALT	LD	Transfer to hospital closer to the patients place of residence
**26**	ALT	Amputation	--
**27**	ALT	LD	--
**28**	ALT	Parascapular	Dermal substitute + skin graft
**29**	FIBU	ALT	Delayed local random pattern flap
**30**	ALT	ALT	Free LD Flap
**31**	RA	LD	--
**32**	LD	Split thickness skin graft	--
**33**	Gracilis	ALT	--
**34**	ALT	LD	--
**35**	Serratus	ALT	--
**36**	LD	LD-PARA	--
**37**	ALT	LD	--
**38**	Parascapular	Parascapular	--
**39**	SCIA	Split thickness skin graft	--
**40**	ALT	Parascapular	--
**41**	LD	Local perforator propeller flap	--
**42**	ALT	LD	--
**43**	ALT	LD	--

LD: Latissimus dorsi; PARA: parascapular; ALT: anterolateral thigh; RA: rectus abdominis; SCIA: superficial circumflex iliac artery flap.

**Table 3:** Surgical strategy for secondary reconstruction.

Patient	Secondary Free Flap	Interval Until Secondary Free Flap (Days)	Recipient Artery	Recipient Vein	Anastomosis A/V	VG	Change in Surgical Strategy	Complications	Outcomes	Cause of Failure
**1**	--	--	--	--	--	--	Amputation	--	Healed stump	--
**2**	--	--	--	--	--	--	Split thickness skin graft	--	Healed wound	--
**3**	ALT	40	ATA	VC	EE/EE	--	Arterial anastomosis	--	SFFT	--
**4**	--	--	--	--	--	--	Amputation	--	Healed stump	--
**5**	Parascapular	32	ATA	VS	ES/EE	--	1. Flap 2. Recipient artery	--	SFFT	--
**6**	--	--	--	--	--	--	Amputation	--	Healed stump	--
**7**	Parascapular	19	ATA	VC	ES/EE	--	1. Flap 2. Arterial anastomosis	--	SFFT	--
**8**	LD	7	ATP	VS	ES/EE	--	Flap	Necrosis of skin graft	SFFT after revision	--
**9**	--	--	--	--	--	--	Matriderm + skin graft	--	Healed wound	--
**10**	LD	15	ATP	VC	EE/EE	--	1. Flap 2. Arterial anastomosis	--	SFFT	--
**11**	LD	29	AFs	VS	EE to loop/EE	AV Loop	1. Recipient artery 2. AV Loop 3. Arterial anastomosis	Venous insufficiency	SFFT after microsurgical revision	--
**12**	Parascapular	13	ATP	VC	ES/EE	--	1. Flap 2. Recipient vein	--	SFFT	--
**13**	LD	7	ATP	VC	ES/EE	--	Flap	--	SFFT	--
**14**	ALT	16	ATP	VC	ES/EE	--	--	--	SFFT	--
**15**	LD	12	AFs	VS	EE to loop/EE	AV Loop	1. Rlap 2. AV loop 3. Recipient artery 4. Arterial anastomosis	--	SFFT	--
**16**	LD	16	ATP	VC	EE/EE	--	Flap	Dehiscence	SFFT after revision	--
**17**	RA	7	ATP	VS	ES/EE	--	Flap	V. Thrombosis	SFFT after microsurgical revision	--
**18**	LD	13	ATA	VC	ES/EE	--	Flap	--	SFFT	--
**19**	--	--	--	--	--	--	Amputation	--	Healed stump	--
**20**	--	--	--	--	--	--	Amputation	--	Healed stump	--
**21**	--	--	--	--	--	--	Amputation	--	Healed stump	--
**22**	LD	18	ATA	VC + VS	EE/EE	--	1. Flap 2. Arterial anastomosis 3. Recipient vein	--	SFFT	--
**23**	--	--	--	--	--	--	Transfer to hospital closer to the patients place of residence	--	Wound remained	--
**24**	Radial forearm	4	ATP	VC	EE/EE	--	1. Flap 2. Arterial anastomosis	A. Thrombosis	Total failure of free flap	A. Thrombosis
**25**	LD	8	--	--	--	--	Flap	Compromised arterial perfusion in situ after dissection	Flap was not transferred.	Not clear
**26**	--	--	--	--	--	--	Amputation	--	Healed stump	--
**27**	LD	25	AFs	VS	EE to loop/EE	AV Loop	1. Flap 2. Recipient artery 3. AV loop	--	SFFT	--
**28**	Parascapular	20	AFs	VS	ES/EE	--	1. Flap 2. Recipient artery	A.V. Thrombosis	Total failure of free flap	A.V. Thrombosis
**29**	ALT	4	ATP	VC	EE to loop/EE	AV Loop	Flap	Intraop arterially insufficient	Total failure of free flap	In-flap thrombosis
**30**	ALT	3	ATP	VC	ES/EE	--	1. Recipient artery 2. Arterial anastomosis	Arterially insufficient	Total failure of free flap	Not clear
**31**	LD	10	ATP	VC	ES/EE	--	1. Flap 2. Recipient vein	Infection	SFFT after débridement.	--
**32**	--	--	--	--	--	--	Split thickness skin graft	--	Healed wound	--
**33**	ALT	12	ATA	VS	ES/EE	--	1. Flap 2. Recipient vein	--	SFFT	--
**34**	LD	8	AFs	VS	EE to loop/EE	AV Loop	Flap	--	SFFT	--
**35**	ALT	25	ATP	VC	ES/EE	--	1. Flap 2. Recipient artery	--	SFFT	--
**36**	LD-PARA	26	Afs	VS	EE to loop/EE	AV-Loop	1. Flap 2. AV loop 3. Arterial anastomosis	--	SFFT	--
**37**	LD	25	ATP	VC	ES/EE	--	1. Flap 2. Arterial anastomosis	--	SFFT	--
**38**	Parascapular	55	Afs	VS	EE to loop/EE	AV-Loop	1. Flap 2. AV loop	--	SFFT	--
**39**	--	--	--	--	--	--	Split thickness skin graft	--	Healed wound	--
**40**	Parascapular	7	Afs	VS	EE to loop/EE	AV-Loop	1. Flap 2. AV loop	--	SFFT	--
**41**	--	--	--	--	--	--	Local perforator propeller flap	--	Healed wound	--
**42**	LD	8	ATA	VC	EE/EE	--	1. Flap 2. Arterial anastomosis	--	SFFT	--
**43**	LD	4	Afs	VS	EE to loop/EE	AV-Loop	Flap	--	SFFT	--

VG: venous grafting; LD: latissimus dorsi; ALT: anterolateral thigh; ATA: arteria tibialis anterior; ATP: arteria tibialis posterior; Afs: arteria femoralis superficialis; VS: vena superficialis; VC: venae comitantes; ES: end to side; EE: end to end; and SFFT: successful free flap transfer.

**Table 4:** Surgical strategy after failure of the secondary reconstruction.

Patient	Tertiary Free Flap	Recipient Artery	Recipient Vein	Anastomosis	VG	Change in Surgical Strategy	Complications	Outcomes	Cause of Failure
**24**	--	--	--	--	--	Delayed reversed sural flap	--	Successful flap transfer	--
**25**	--	--	--	--	--	Transfer to hospital closer to the patients place of residence	--	Wound remained	--
**28**	--	--	--	--	--	Dermal substitute + skin graft	--	Healed wound	--
**29**	--	--	--	--	--	Delayed local random-pattern flap	--	Successful flap transfer	--
**30**	LD	ATP	VC	ES/EE	--	Free LD Flap	--	Successful free flap transfer	--

VG: venous grafting; LD: latissimus dorsi; ALT: anterolateral thigh; ATP: arteria tibialis posterior; VS: vena superficialis; VC: venae comitantes; ES: end to side; EE: end to end; NPWT: negative pressure wound therapy.

## Data Availability

The data presented in this study are available on request from the corresponding author. The data are not publicly available due to local privacy laws.
